# Assessment of search strategies in Medline to identify studies on the impact of long COVID on workability

**DOI:** 10.3389/frma.2024.1300533

**Published:** 2024-03-01

**Authors:** Jean-François Gehanno, Isabelle Thaon, Carole Pelissier, Laetitia Rollin

**Affiliations:** ^1^Institute of Occupational Medicine, Rouen University Hospital, Rouen, France; ^2^Inserm, Rouen University, Sorbonne University, University of Paris 13, Laboratory of Medical Informatics and Knowledge Engineering in e-Health, LIMICS, Paris, France; ^3^Centre de Consultations de Pathologie Professionnelle, CHRU de Nancy, Vandoeuvre les Nancy, Nancy, France; ^4^Centre Hospitalier Universitaire de Saint-Etienne, Université Lyon 1, Université de St Etienne, Université Gustave Eiffel-IFSTTAR, Saint-Etienne, France; ^5^UMRESTTE UMR-T9405, Saint-Etienne, France

**Keywords:** long COVID, work, MEDLINE, bibliometrics, information retrieval methods

## Abstract

**Objectives:**

Studies on the impact of long COVID on work capacity are increasing but are difficult to locate in bibliographic databases, due to the heterogeneity of the terms used to describe this new condition and its consequences. This study aims to report on the effectiveness of different search strategies to find studies on the impact of long COVID on work participation in PubMed and to create validated search strings.

**Methods:**

We searched PubMed for articles published on Long COVID and including information about work. Relevant articles were identified and their reference lists were screened. Occupational health journals were manually scanned to identify articles that could have been missed. A total of 885 articles potentially relevant were collected and 120 were finally included in a gold standard database. Recall, Precision, and Number Needed to Read (NNR) of various keywords or combinations of keywords were assessed.

**Results:**

Overall, 123 search-words alone or in combination were tested. The highest Recalls with a single MeSH term or textword were 23 and 90%, respectively. Two different search strings were developed, one optimizing Recall while keeping Precision acceptable (Recall 98.3%, Precision 15.9%, NNR 6.3) and one optimizing Precision while keeping Recall acceptable (Recall 90.8%, Precision 26.1%, NNR 3.8).

**Conclusions:**

No single MeSH term allows to find all relevant studies on the impact of long COVID on work ability in PubMed. The use of various MeSH and non-MeSH terms in combination is required to recover such studies without being overwhelmed by irrelevant articles.

## Introduction

The definition of post-COVID or long-COVID is the presence of symptoms that last for at least 2 months, cannot be explained by an alternative diagnosis, and occurs usually 3 months after a history of probable or confirmed SARS CoV-2 infection (Soriano et al., 2022). Among those who had symptomatic SARS-CoV-2 infection, it has been estimated that 6.2% (2.4–13.3%) experience at least one long COVID symptom (Global Burden of Disease Long COVID Collaborators, 2022). This condition affects mostly people aged 20 years or older, and among individuals with Long COVID symptoms 3 months after symptomatic SARS-CoV-2 infection, 15% continue to experience symptoms at 12 months (Global Burden of Disease Long COVID Collaborators, 2022). A growing body of evidence outlines the impact of this new condition on work and occupational health. An Italian study with 15 months of follow-up of 247 patients of working age observed that more than two-thirds of individuals still symptomatic at 200 days had an impaired Work Ability Index (Sansone et al., 2023). A Dutch study found that, on average, 5.5% of employees who contracted COVID-19 were absent for over 12 weeks (Aben et al., 2023).

However, studies in that field are difficult to retrieve since there is a multiplicity of titles used to describe the same clinical problem, such as long COVID, post-COVID symptoms, post-COVID syndrome, post-COVID condition, and post–acute COVID, among others, and no single keyword can effectively cover the entire field (Chen et al., 2022; Pan and Pareek, 2023). Information retrieval is usually achieved by searching bibliographic databases, MEDLINE being the largest and most widely used one (Dunn et al., 2017). It is freely accessible through the PubMed interface on the internet, it is comprehensive, especially for high-quality articles and it provides many tools, including a controlled thesaurus of medical titles, known as medical subject heading (MeSH) terms, which help users recover relevant information (Rollin et al., 2010; Halladay et al., 2015; Sampson et al., 2016).

A literature search should yield as much information as possible on a specific topic, with the fewest articles as possible that are unrelated to the search topic. In bibliometrics science, this translates into a search that has both good Recall (number of relevant retrieved documents over the number of existing relevant document) and high Precision (number of relevant retrieved documents divided by the total number of documents retrieved) ratios, which are comparable to the concepts of sensitivity and specificity in epidemiology.

Many studies on the effectiveness of literature search strategies have been published (Cooper et al., 2018), some of them concerning specifically occupational health (Verbeek et al., 2005; Gehanno et al., 2009; Mattioli et al., 2010, 2012, 2013). These strategies involve the use of MeSH terms or textwords, alone or in combination, to achieve comprehensive yet accurate retrieval. However, since no search strategy is validated to find studies on the impact of long COVID on work, authors of reviews or meta-analyses on this topic used different strategies and keywords, usually without mentioning the effectiveness of their search strings, making their utilization by other potentially irrelevant (Lopez-Leon et al., 2021; Fernández-de-las-Peñas et al., 2022; Veronese et al., 2022; Lai et al., 2023; Tsampasian et al., 2023; Watanabe et al., 2023).

Furthermore, a search strategy should depend on its objectives. When comprehensiveness is needed, for example in systematic reviews or meta-analyses, the search strings used must focus on the highest Recall possible, even if the consequence will be having to go through a large number of irrelevant results. When the purpose is to stay in touch with medical knowledge in this field or to answer a specific question in a limited timescale, a search string privileging Precision will be recommended, even at the price of missing some potentially relevant articles.

Therefore, the purpose of this study was to report on the effectiveness of various search strategies and keywords to identify studies on long COVID and work in the PubMed bibliographic database.

## Methods

We built the gold-standard (GS) database of articles published on return to work or work limitations and Long COVID in two steps. Searches were performed in March 2023.

To recover studies on Long COVID in PubMed, we started with the search string developed by Langnickel et al. (2022), and we added the keywords used in other reviews on Long-COVID (Lopez-Leon et al., 2021; Fernández-de-las-Peñas et al., 2022; Veronese et al., 2022; Lai et al., 2023; Watanabe et al., 2023).

The final search string was (“Post-acute COVID-19 syndrome” OR “Long COVID” OR “Post COVID” OR “Chronic COVID” OR “Persistent COVID” OR ((“COVID-19” OR “2019-nCoV” OR “SARS-CoV-2”) AND (“long-term” OR “persist^*^”[Ti] OR “sequela^*^”[Ti] OR “prolong^*^”[Ti]))). This gave 23,332 results.

To identify among them studies on return to work, we used the search strings developed by Gehanno et al. (2009). Since Long COVID can also impair work participation, apart from return to work, we also used keywords identified in a systematic review of work participation outcomes measured in randomized controlled trials (Ravinskaya et al., 2022). The search string was (“Return to work” OR “rehabilitation, vocational”[MeSH] OR (“occupations”[MeSH] OR “occupations” OR “vocation” OR “vocations” OR “vocational”) OR “Sick Leave”[MeSH] OR “work status” OR “Back to work” OR “rehabilitation”[MeSH Subheading] OR “Work participation”[TiAB] OR “Sick Leave”[TiAB] OR “sick leave”[TiAB] OR “absenteeism”[TiAB] OR “presenteeism”[TiAB] OR “work disability”[TiAB] OR “work disability”[TiAB] OR “work ability”[TiAB] OR “functioning”[TiAB] OR “impairments”[TiAB] OR “productivity”[TiAB] OR “employment”[TiAB] OR “WPAI” OR “WPAS” OR “SF-HLQ” OR “WLQ 25” OR “USSQ” OR “SAS-work” OR “EHP-30” OR “Stanford Presenteeism scale” OR “RA-WIS”).

The combination of the two search strings gave 885 articles.

Two independent examiners looked at each article (title, abstract and full text when needed) to select those matching our criteria of inclusion. Studies were considered relevant when they described work participation or work limitations, including sick leave or disability, for individuals with Long COVID, according to the WHO definition (Soriano et al., 2022). Studies with a follow-up shorter than 3 months or studies focusing only on risk factors for impaired working ability, without directly assessing working status, were excluded. Discrepancies between examiners were resolved by discussion and compromise.

Overall, 85 articles were considered relevant. The screening of their reference lists led us to identify 31 other relevant articles.

In a second step, we hand-searched the tables of content of the 15 occupational health journals indexed in the Journal of Citation Reports and PubMed.

Among the 4,963 articles published by these journals between 2020 and March 2023, 789 concerned COVID-19. Four relevant articles, not identified in previous steps, were recovered.

Overall, the GS database contained 120 articles.

MeSH terms of the articles in the GS were extracted using a Medline Post Processor (Meva). The articles were indexed by a total of 871 MeSH terms, with 197 different terms. Two authors independently selected the MeSH terms considered relevant for work-related studies.

The titles and abstracts of the 120 articles were analyzed by a web-based reading and analysis environment for digital texts (https://voyant-tools.org/). It identified 91,880 words. Among the words that appeared at least 10 times, we selected the ones which were considered relevant for studies concerning work participation, i.e., 26 text words.

Recall and Precision are the main dimensions used to determine the utility of an information retrieval system or a search strategy (Cooper et al., 2018). Precision is the number of relevant documents retrieved by a search divided by the total number of documents retrieved by that search. Recall is similar to the epidemiological concept of sensitivity. A precise search will identify mostly relevant studies, with as few false positive hits as possible but with many false negatives, whereas a search with a high Recall will provide a higher number of results, with a significant proportion of irrelevant articles.

The Number Needed to Read (NNR = 1/Precision) is the same concept as the number needed to treat (NNT). It assesses the number of references that have to be screened to find one of relevance.

We computed the Recall and Precision of the 54 search words (MeSH terms and textwords) identified, and of the 28 MeSH terms. The 82 search words were searched one by one in the PubMed subset of 22,445 articles on long COVID and the results were compared to the GS database using the PubMed Advanced Search Builder (https://pubmed.ncbi.nlm.nih.gov/advanced/) to assess the Recall and Precision of all of them.

To compute Recall, search words which had a Recall higher than 10% were added one by one in different search strings.

To compute Precision, search words with a Precision of at least 10% and Recall of more than 10% were added one by one by in different search strings.

The result of each of these search strings was compared to the GS database to assess true positives, false positives and false negatives hits.

The effectiveness of a literature search is not clearly defined and studies performed to elaborate “effective” searches usually do not mention which thresholds, for Recall and Precision, were used (Cooper et al., 2018). Effectiveness depends on the objectives of the search, i.e., if comprehensive results are needed or if the time spent in searching must be short.

For these two objectives, thresholds for Recall of 90% with a minimum Precision of 5% (NNR ≤ 20), or 65% for Recall, with a minimum Precision of 20% (NNR ≤ 5) are commonly accepted (Verbeek et al., 2005; Schaafsma et al., 2006; Kok et al., 2015; Cooper et al., 2018). These thresholds were used in our study.

All the results (Recall, Precision, and NNR) for each search word or combination were entered in an Excel file and analyses were performed with Excel (Microsoft).

## Results

The final GS database gathered the 120 articles which were considered relevant. They had been published in 78 different journals and were described by 197 different MeSH terms. Some of these MeSH terms were generic (e.g., gender, type of study or age class) and were not used to build search strings. The combination of the two MeSH terms that could be considered to describe the most adequately our topic, i.e., “Post acute covid 19 syndrome”[MeSH] AND “Work”[MeSH], had a Precision of 75.00% but a Recall of only 7.50%. Concerning specific descriptors, the best single terms alone or in combination, combined with the search string on long COVID, either in terms of Recall or in terms of Precision, are presented in Tables 1, 2. Considering only relevant MeSH terms, the single MeSH term with the highest recall was “Socioeconomic Factors”, with a recall of 23.33% and a precision of 10.69%. The single textword with the highest recall was “work^*^”, with a recall of 90% and a precision of 3.93%. Some search words provided different results if they were searched as MeSH terms or as textwords in Title/Abstract. For example, searching “Work” as a MeSH term or as a textword in Title/Abstract found 16 and 100 articles from the GS database, respectively. Combining both found 102 articles, which gave a Recall of 85.00% and a Precision of 36.17%.

**Table 1:** Best search strings, in terms of Recall (>95%), to retrieve articles on the impact of long COVID on work.

**Search**	**Search terms^a^**	**Precision**	**Recall**	**NNR**
A1	(“Work^*^”[TiAB] OR “Fatigue”[TiAB] OR “Occupation^*^”[TiAB] OR “Socioeconomic Factors”[MeSH] OR “Return^*^”[TiAB] OR “Rehabilitation”[TiAB])	16%	98%	6.3
A2	(“Work^*^”[TiAB] OR “Fatigue”[TiAB] OR “Occupation^*^”[TiAB] OR “Return to Work”[MeSH] OR “Disability”[TiAB])	19%	98%	5.1
A3	(“Work^*^”[TiAB] OR “Fatigue”[TiAB] OR “Occupation^*^”[TiAB] OR “Socioeconomic Factors”[MeSH] OR “Return^*^”[TiAB])	19%	98%	5.2
A4	(“Work^*^”[TiAB] OR “Fatigue”[TiAB] OR “Occupation^*^”[TiAB] OR “Socioeconomic Factors”[MeSH] OR “Return^*^”[TiAB] OR “Sick Leave”[TiAB])	19%	98%	5.2
A5	(“Work^*^”[TiAB] OR “Fatigue”[TiAB] OR “Occupation^*^”[TiAB] OR “Employment”[MeSH] OR “Return to Work”[MeSH])	21%	97%	4.8
A6	(“Work^*^”[TiAB] OR “Fatigue”[TiAB] OR “Occupation^*^”[TiAB] OR “Return to Work”[MeSH])	21%	97%	4.8
A7	(“Work^*^”[TiAB] OR “Fatigue”[TiAB] OR “Occupation^*^”[TiAB] OR “Employment”[MeSH])	20%	97%	5.0
A8	(“Work^*^”[TiAB] OR “Fatigue”[TiAB] OR “Occupation^*^”[TiAB] OR “Return to Work”[MeSH])	20%	97%	5.0
A9	(“Work^*^”[TiAB] OR “Fatigue”[TiAB] OR “Occupation^*^”[TiAB] OR “Socioeconomic Factors”[MeSH])	20%	97%	5.0
A10	(“Work^*^”[TiAB] OR “Fatigue”[TiAB] OR “Occupation^*^”[TiAB] OR “Return^*^”[TiAB] OR “Sick Leave”[TiAB])	20%	97%	5.1
A11	(“Work^*^”[TiAB] OR “Fatigue”[TiAB] OR “Occupation^*^”[TiAB] OR “Employment”[MeSH] OR “Return to Work”[MeSH] OR “Outcome^*^”[TiAB])	16%	97%	6.3
A12	(“Work^*^”[TiAB] OR “Fatigue”[TiAB] OR “Occupation^*^”[TiAB])	21%	96%	4.9

^a^Combined with the search string on long COVID.

NNR, Number Needed to Read.

^*^Truncation.

**Table 2:** Best search strings, in terms of Precision (>25%), to retrieve articles on the impact of long COVID on work.

**Search**	**Search terms^a^**	**Precision**	**Recall**	**NNR**
B1	(“Work”[MeSH] OR “Work”[TiAB])	36%	85%	2.8
B2	(“Work^*^”[TiAB] OR “Return”[TiAB])	26%	91%	3.8
B3	(“Work^*^”[TiAB] OR “Return^*^”[TiAB])	25%	91%	4.0

^a^Combined with the search string on long COVID.

NNR, Number Needed to Read.

^*^Truncation.

If a high Recall is needed, the search string A1 (Recall 98.3%) is the best, but at the price of an NNR of 6.3 (Table 1).

The best compromise between Recall and Precision is given by the search string B2 (Table 2), with a Recall of 90.8% and a Precision of 25.2% (NNR 3.8).

## Discussion

No single MeSH term can efficiently identify studies on the impact of long COVID on work in PubMed, and it requires different combinations of MeSH terms and textwords. We developed two search strings that allow a high Recall or a compromise between Recall and Precision.

We built our GS with a “snowball method”, combining PubMed searches and hand searching, as in previous studies on the effectiveness of literature searching (Gehanno et al., 2009, 2023), whereas many studies on that topic relied on hand searching only in a limited, and therefore unsystematic, number of journals. This last approach underestimates the number of publications in PubMed as a whole and provides fewer results than the use of PubMed (Verbeek et al., 2005; Mattioli et al., 2012; Cooper et al., 2018).

Such an objectively derived strategy allows to collect more comprehensively relevant articles (Hausner et al., 2012), and our GS database may be considered to be as exhaustive as possible.

A weakness of our study concerns the first phase, in which we built a first database containing potential studies on long COVID since there is no validated strategy to identify such articles in PubMed. Nevertheless, we used many different keywords, favoring Recall over Precision, and we obtained nearly twice as many articles than those on long COVID indexed in LitCovid, a resource created by the National Library of Medicine and providing central access to relevant articles in PubMed.

Another weakness could rely on the high proportion of articles we excluded from the 885 identified by combining the search strings on long COVID and work. However, it has been observed that published studies on long COVID use different definitions, many of them not complying with the definitions from the WHO, NICE, or CDC (Chaichana et al., 2023).

Finally, the search strings we developed are effective for PubMed, but this database does not embrace the total occupational health literature. When comprehensiveness is required, searching also in Embase or PsycInfo may be necessary (Haafkens et al., 2006).

Since the maximum Recall that can be achieved using only MeSH terms is not more than 50%, strategies combining textwords and MeSH terms are recommended to achieve comprehensiveness when searching for studies on the impact of long COVID on workability.

This study identified two different search strategies that can be used by researchers or by healthcare workers to answer questions raised during their daily practice.

For researchers, or in circumstances where comprehensiveness is needed, Recall must be at least 90%, with a minimum Precision of 5% (NNR ≤ 20) (Verbeek et al., 2005; Schaafsma et al., 2006; Kok et al., 2015). In our study, we were able to reach a Recall of 98.3%% while keeping the NNR lower than 20 (Search string A1, Table 1).

For healthcare workers who have a limited amount of time to search for evidence and to separate the wheat from the chaff, recommended thresholds are 65% for Recall and 20% for Precision (Schaafsma et al., 2006; Cooper et al., 2018). In our study, the best compromise between Recall and Precision was given by the combination B2, with a Recall of 90.83% and a Precision of 26.01% (NNR 3.8).

However, efforts should be made to reduce the multiplicity of denominations used to describe long COVID, to more easily identify relevant studies.

Moreover, studies assessing Precision and Recall of search strategies on long COVID in different databases would be of interest considering the increasing research on physiopathology and consequences of this new disease.

## Conclusion

Identifying relevant studies concerning the impact of long COVID on work in PubMed is complex and cannot be achieved by using MeSH terms only. Obtaining a satisfying Recall, without sacrificing Precision necessitates complex combinations of MeSH terms and textwords. However, it is necessary to decide what thresholds should be used for Precision and Recall, according to the results that are expected, i.e., thoroughness or expeditiousness, before starting searching to be as efficient as possible.

## Data availability statement

The datasets presented in this study can be found in online repositories. The names of the repository/repositories and accession number(s) can be found below: https://www.ncbi.nlm.nih.gov/sites/myncbi/1XUp-adEoi/collections/62707925/public/.

## Author contributions

J-FG: Conceptualization, Data curation, Formal analysis, Investigation, Methodology, Project administration, Validation, Writing – original draft, Writing – review & editing. IT: Data curation, Formal analysis, Investigation, Methodology, Writing – review & editing. CP: Data curation, Formal analysis, Investigation, Writing – review & editing. LR: Conceptualization, Data curation, Formal analysis, Investigation, Methodology, Supervision, Validation, Writing – review & editing.
